# Aminated Graphene-Graft-Oligo(Glutamic Acid) /Poly(ε-Caprolactone) Composites: Preparation, Characterization and Biological Evaluation

**DOI:** 10.3390/polym13162628

**Published:** 2021-08-07

**Authors:** Mariia Stepanova, Olga Solomakha, Maxim Rabchinskii, Ilia Averianov, Iosif Gofman, Yuliya Nashchekina, Grigorii Antonov, Aleksey Smirnov, Boris Ber, Aleksey Nashchekin, Evgenia Korzhikova-Vlakh

**Affiliations:** 1 Institute of Macromolecular Compounds, Russian Academy of Sciences, 199004 St. Petersburg, Russia; maristepanova@hq.macro.ru (M.S.); solomanya@bk.ru (O.S.); aaardvark@yandex.ru (I.A.); gofman@imc.macro.ru (I.G.); 2 Ioffe Institute, Politekhnicheskaya st. 26, 194021 St. Petersburg, Russia; antonov@scamt-itmo.ru (G.A.); sab@mail.ioffe.ru (A.S.); boris.ber@mail.ioffe.ru (B.B.); nashchekin@mail.ioffe.ru (A.N.); 3 Institute of Cytology, Russian Academy of Sciences, 194064 St. Petersburg, Russia; ulichka@mail.ru

**Keywords:** aminated graphene, graphene modification, grafting from, oligomers of glutamic acid, poly-ε-caprolactone, biocompatible polymer composites

## Abstract

Biodegradable and biocompatible composites are of great interest as biomedical materials for various regeneration processes such as the regeneration of bones, cartilage and soft tissues. Modification of the filler surface can improve its compatibility with the polymer matrix, and, as a result, the characteristics and properties of composite materials. This work is devoted to the synthesis and modification of aminated graphene with oligomers of glutamic acid and their use for the preparation of composite materials based on poly(ε-caprolactone). Ring-opening polymerization of N-carboxyanhydride of glutamic acid γ-benzyl ester was used to graft oligomers of glutamic acid from the surface of aminated graphene. The success of the modification was confirmed by Fourier-transform infrared and X-ray photoelectron spectroscopy as well as thermogravimetric analysis. In addition, the dispersions of neat and modified aminated graphene were analyzed by dynamic and electrophoretic light scattering to monitor changes in the characteristics due to modification. The poly(ε-caprolactone) films filled with neat and modified aminated graphene were manufactured and carefully characterized for their mechanical and biological properties. Grafting of glutamic acid oligomers from the surface of aminated graphene improved the distribution of the filler in the polymer matrix that, in turn, positively affected the mechanical properties of composite materials in comparison to ones containing the unmodified filler. Moreover, the modification improved the biocompatibility of the filler with human MG-63 osteoblast-like cells.

## 1. Introduction

Currently, aliphatic polyesters are the widely used class of biodegradable polymers for the preparation of various biomedical materials [1,2,3]. The application of poly(lactic acid) (PLA), poly(glycolic acid-co-lactic acid) (PLGA), poly(ε-caprolactone) (PCL) and polyhydroxybutyrate (PHB) in medicine is due to their biocompatibility with human tissues and their ability to biodegrade to nontoxic metabolites [4]. In addition to their applications as drug delivery systems [3] and surgical suture threads [5], they are widely studied as materials for regenerative medicine including scaffolds for bone tissue engineering [6,7]. However, the hydrophobic properties of aliphatic polyesters impair the adhesion of cells while the lack of a sufficient number of the reactive groups limits the possibility of modifying those polymers with bioactive molecules [8,9]. Moreover, the mechanical properties of neat materials based on aliphatic polyesters should be improved so that they can be considered as scaffolds for bone regeneration [10,11].

Among the strategies allowing the regulation of properties of a polymer material, the preparation of biocomposites via the incorporation of biofunctional and/or reinforcing nano- or microparticles in the polymer matrix can be a matter of choice [10,12]. Such particles could improve the mechanical properties [11,13,14] or create the time–spatial distribution of biological ligands [15,16], while the polymer matrix would be degraded in vivo. Moreover, the ability to influence mineralization is one of the key properties of the resulting materials for bone tissue regeneration [16,17]. In this regard, hydroxyapatite [18] or ceramic nanoparticles [19] are often used as fillers both to improve mechanical properties and induce the mineralization of implanted materials.

Recently, it was shown that the application of graphene as a filler can improve the mechanical properties of polymer materials [20,21]. The use of graphene derivatives such as graphene oxide (GO) or reduced graphene oxide (rGO), which contain hydroxylic and carboxylic groups, can also improve the interaction of composites with cells and biomolecules and enhance cell growth, cell differentiation and cell proliferation [22,23]. At the same time, GO and rGO similar to graphene enhances the mechanical properties of polymer materials [24,25]. Recently, Krystyjan et al. and Mohamad et al. prepared the starch/chitosan [26] and chitosan/PLA-based [27] composites containing GO as filler, respectively. In both cases, the addition of GO improved the mechanical properties of polymer materials. Furthermore, the developed polymer composites containing GO showed bacteriostatic activity but were not toxic to human cells. Luo et al. have reported that GO incorporated on electrospun PLGA demonstrated osteoinductive properties when compared to the scaffolds without GO [28].

One of the main drawbacks in preparing of many composites is the aggregation of the fillers in a polymer matrix [29,30]. To overcome this obstacle, many studies have focused on modifying fillers to increase their phase compatibility and homogeneous dispersion state. Surface grafting with oligomers or polymers is a well-known approach to improve the filler distribution in the polymer matrix [31,32,33]. Recently, we have successfully applied this approach to improve the compatibility of the hydrophobic PLA and PCL with hydrophilic filler (nanocrystalline cellulose) by its grafting with amphiphilic polypeptide [32]. Such modification allowed the enhancement of the composite mechanical characteristics and also favored the matrix mineralization [12,34].

In the case of graphene-based materials, the strong π–π interaction contributes to a pronounced aggregation of graphene and, as a result, its poor distribution in the polymer matrix. Partly, this effect can be overcome by the use of GO or rGO. Furthermore, GO and rGO can give more stable dispersions in water and polar solvents than graphene. In particular, Gu et al. reported the modification of GO with polyamide in ethanol followed by melt spinning [35]. The developed composites provided excellent mechanical properties. Wang et al. proposed a modification of GO with poly(lactic acid) to adjust GO surface properties and enhance phase compatibility with polymer matrix (PLA) [36].

The goal of this study was the development of a technique for the modification of aminated rGO (rGO-Am) with oligomers of glutamic acid (oligo(Glu)) as a moiety with osteoinductive properties [16]. The grafting of oligo(Glu) from the rGO-Am surface was performed by the ring-opening polymerization of N-carboxyanhydride of γ-benzyl ester of glutamic acid initiated by primary amino groups of rGO-Am. The modification of rGO-Am was carefully tested by a number of physicochemical methods. Neat and modified rGO-Am was applied as a filler to prepare PCL-based biocompatible composite films whose mechanical and biological properties were investigated and compared. The biocompatibility of dispersions of the fillers as well as composite films with human MG-63 osteoblast-like cells was studied in order to assess the applicability of the developed composites as potential materials for bone tissue regeneration.

## 2. Materials and Methods

### 2.1. Materials

An aqueous dispersion of GO was purchased from Graphene Technologies (Moscow, Russia, www.graphtechrus.com accessed on 30 June 2019). γ-benzyl-L-glutamate (Glu(OBzl)) (>99%), triphosgene (98%) and α-pinene (98%) used for the synthesis of N-carboxyanhydride as well as *n*-hexylamine (98%) used as an initiator were purchased from Sigma-Aldrich (Darmstadt, Germany). All organic solvents used in this work were purchased from Vecton Ltd. (St. Petersburg, Russia) and distilled before use.

Low-molecular-weight poly(glutamic acid) (PGlu) used for comparison was synthesized in IMC RAS as described earlier [32] and had the following characteristics according to size-exclusion chromatography (SEC): *M*_w_ = 10,800, *M*_n_ = 7700 and Đ = 1.40. According to ^1^H NMR, the used PGlu sample contained 15% of residual benzyl groups (PGlu(OBzl)).

Membranes for dialysis with molecular weight cutoff (MWCO) 1000, 3500, 6000−8000 and 12,000–14,000 were purchased from Orange Scientific (OrDialDClean regenerated cellulose dialysis tubing, Anaheim, CA, USA). Vivaspin concentrators used for ultrafiltration were products of Sartorius (Göttingen, Germany).

Human MG-63 osteoblast-like cell line was obtained from the Vertebrate Cell Culture Collection (Institute of Cytology RAS, St. Petersburg, Russia). They were cultured at 37 °C in humidified 5% CO_2_ and cultured in EMEM (Lonza, St. Louis, MO, USA) containing 10% fetal bovine serum (FBS; HyClone, Logan, UT, USA), 1% NEAA (FBS; HyClone, Logan, UT, USA) and 1% penicillin/streptomycin (Sigma-Aldrich, Darmstadt, Germany). In all, 4~6 passage cells were used for this study. On attaining 80–90% confluency, the cells were trypsinized with trypsin-EDTA (Sigma-Aldrich, Darmstadt, Germany).

### 2.2. Synthesis of Aminated Graphene

The synthesis of the rGO-Am was carried out according to the previously described method [37]. In brief, GO suspension of 0.05 wt.% concentration was centrifuged at 18,100 g for 20 min (Sigma S-16 centrifuge, Sigma-Aldrich, Darmstadt, Germany), the supernatant was decanted away, and the sediment was transferred into a fluoroplastic cup and rinsed with 46% HBr (Sigma-Aldrich, Darmstadt, Germany). The obtained suspension was further heated at 80 °C for 48 h in air while stirring. Afterward, the synthesized intermediate (brominated graphene) was copiously washed by isopropyl alcohol using a glass filter (of 40 μm of pore size), the sediment was placed into a fluoroplastic cup, and rinsed with a saturated solution of ammonia in isopropyl alcohol. The obtained suspension was stirred for 24 h, resulting in the formation of rGO-Am. To purify the synthesized rGO-Am from the residuals of the reaction mixture, it was washed several times with isopropyl alcohol using a glass filter (of 40 μm of pore size).

### 2.3. Modification of Aminated Graphene with Oligomers of Glutamic Acid

Two milliliters of distilled and dried ethyl acetate were added to a glass Schlenk tube containing 110 mg of rGO-Am and the mixture was ultrasonicated with an ultrasonic probe UP 50H Hielscher Ultrasonics (Hielscher, Teltow, Germany) at 15–20% power for 1 min. The obtained dispersion was purged with argon for 3 min.

N-carboxyanhydride of γ-benzyl ester of glutamic acid (Glu(OBzl) NCA; monomer) was prepared using a standard protocol as described elsewhere [38,39]. Then, 230 mg of monomer was dissolved in 2 mL of dry ethyl acetate and the solution was purged with argon for 10 min. The resulting solution was added to the rGO-Am dispersion, ultrasonicated with an ultrasonic probe for 1–2 min, sealed, again purged with argon for 10 min, and incubated at 35 °C for 2 days. After this, 5 mL of DMF was added to the reaction mixture. The dispersion was ultrasonicated in the ultrasound bath for 2 min and finally centrifuged at 15,300 g for 15 min. After the removal of the supernatant, a fresh portion of DMF (5 mL) was added to the sediment, and the procedure of redispersion and centrifugation was repeated again. The washing procedure was repeated five times. Finally, the modified rGO-Am was redispersed in 7 mL of methanol, centrifuged as described above, and the sediment was dried in a vacuum at 22 °C for 12 h. The product weight was 103 mg (~30%).

To remove the benzyl protective groups, 2 mL of the DMSO/TFA (1/1, *v/v*) mixture was added to 50 mg of rGO-Am-oligo(Glu(OBzl)). The reaction was carried out in an ice bath (0–5 °C) for 1 h. After this time, the ice bath was removed, 50 mL of DMSO/TFMSA solution (4/1, *v/v*) was added, and the dispersion was stirred for additional 1 h at 22 °C. After that, 6 mL of DMF was added to the resulting dispersion and dialysis against water using a membrane bag with MWCO = 1000 was carried out for 5 days. The purified product was freeze-dried and stored at 4 °C before use. The product weight was 33 mg (~66%).

### 2.4. Characterization of rGO-Am and Its Derivatives

The survey, C 1s, N 1s and O 1s X-ray photoelectron spectra (XPS) were measured using a Thermo Fisher ESCALAB 250Xi XPS system (Thermo Fisher Scientific, Waltham, MS, USA) with a monochromatic Al Kα X-ray source (*hv* = 1486.68 eV). For all the spectroscopic measurements, the studied materials were deposited onto the silicon wafers by the drop-casting of 20–30 μL of the corresponding isopropyl suspension, of 5 × 10^−1^ wt.% of concentration with subsequent drying overnight at room temperature. Prior to the measurements, samples were evacuated down to a pressure *P* = 10^−9^ Torr at least for 20 h to remove all adsorbates. The spectra were calibrated with respect to the Au 4f7/2 line (84.0 eV).

CasaXPS© software (Version 2.3.16Dev52, Casa Software Ltd.; Teignmouth, United Kingdom) was used for the deconvolution and quantification of the acquired C 1s, N 1s and O 1s X-ray photoelectron spectra. All the spectra were fitted with a Shirley background. In the case of C 1s spectra, a set of one asymmetric Doniach-Sunjic function ((DS0.10,100)(GL90)) and six symmetric Gaussian−Lorentzian convoluted functions of 70–30% ratio (GL(30)) was applied. At the same time, three symmetric Gaussian−Lorentzian converged functions of 70–30% ratio (GL(30)) were used for the deconvolution of the N 1s and O 1s spectra. The χ^2^ minimization was ensured using the nonlinear least squares routine. Afterward, the C/O ratios and the relative concentration of the carbon atoms in different states were calculated.

Fourier-transform infrared spectroscopy (FTIR) was performed using IRAffinity-1 S Shimadzu (Shimadzu, Kyoto, Japan). The spectra were recorded for 0.2 mg of sample evenly distributed in 20 mg in a KBr tablet.

The hydrodynamic diameter and electrokinetic potential of neat and modified rGO-Am particles were determined by dynamic and electrophoretic light scattering (DLS and ELS, respectively). All measurements were performed in water at concentration of particles equal to 0.5 mg/mL using a Zetasizer Nano-ZS (Malvern Instruments, Malvern, United Kingdom) equipped with a He–Ne laser at 633 nm at scattering angles of 173° and 25°.

The morphology of the studied materials was evaluated with scanning electron microscopy (SEM) using JSM-7001F Jeol microscope (Jeol Ltd., Tokyo, Japan). Optical microscopy in transmitted and reflected light was carried out with the use of the Nikon Eclipse E200 microscope (Nikon Corp., Tokyo, Japan).

Thermogravimetric analysis (TGA) was performed with the use of a DTG-60 Shimadzu (Shimadzu, Kyoto, Japan) in an air atmosphere at a constant heating rate of 5 °C/min. The analysis was performed in the temperature range from 40 to 600 °C using the pre-homogenized samples.

### 2.5. Synthesis of PCL

Polymerization of ε-caprolactone was carried out in bulk at 130 °C for 20 h. Before polymerization, the weighted portion of the monomer was placed into a Schlenk flask and purged with argon for 10 min. The ratio of monomer to stannous octoate was 3600. After synthesis, the polymer was dissolved in a minimum amount of chloroform and precipitated into cold methanol. The precipitated polymer was dried in a vacuum (ca. 150 Pa). PCL yield was 91%.

The molecular weight characteristics (*M*_w_ and *M*_n_) and dispersity (*Đ*) of the polymer obtained were determined by SEC with the use of a Shimadzu HPLC system (Shimadzu, Kyoto, Japan) consisting of a pump LC-10AD VP, system controller SCL-10A VP, and refractometric detector RID-10A (Shimadzu, Canby, OR, USA) supplied with a Rheodyne 725i injection valve (Rohnert Park, CA, USA) and two columns of Agilent PLgel MIXED-D (7.5 × 300 mm, 5 μm) (Agilent, Santa-Clara, CA, USA). The analysis was carried out in THF at a temperature of 40 °C, a flow rate of the mobile phase of 1.0 mL/min. Calculations of molecular weight were fulfilled regarding polystyrene standards with molecular weights in the range of 2000–450,000. Data processing was performed using LC Solution Shimadzu software (version 1.25, Shimadzu, Kyoto, Japan). In addition, the intrinsic viscosity (η) of the synthesized PCL was calculated after measurements of viscosity for a set of solutions with different concentrations in CHCl_3_ using Ostwald’s capillary viscosimeter.

### 2.6. Manufacturing of Composite Films

The pure and composite films based on PCL were manufactured using the previously developed procedure for the PCL composite films with nanocrystalline cellulose [12]. Briefly, a 5% PCL solution in CHCl_3_ (6.5 mL) was poured inside the glass cylinder (i.d. = 75 mm) with fixed cellophane bottom. The solution was left for 12 h in the air for chloroform evaporation. After cellophane removal, the obtained PCL-based films were dried in the air thermostat at 45 °C for 3 days. In the case of composite films, unmodified or modified rGO-Am was dispersed in PCL solution in chloroform and shortly ultrasonicated with the ultrasonic probe for 15–20 s at 15–20% power. The amount of a filler was equal to 0.5 and 1.0 wt.%. For neat rGO-Am, 3 wt.% of the filler was used also for comparison. Other manipulations were done as described above for the manufacturing of unfilled PCL films.

### 2.7. Mechanical Testing

Mechanical properties of the films were studied under uniaxial extension using band-like specimens of 2 mm × 20 mm using the AG-100kNX Plus Shimadzu universal mechanical system (Shimadzu, Kyoto, Japan). The thickness of the tested specimens was 90 ± 10 µm. The extension speed was 10 mm/min. The characteristics of the tested samples were calculated basing on the results of measurements for 7–11 fragments of the material and the results are given as average value ± SD.

### 2.8. Biological Evaluation

The MTT assay was employed to assess the cell viability and proliferation of the MG-63 on interaction with the material. This is a colorimetric assay measuring the reduction of yellow 3-(4,5-dimethythiazol-2-yl)-2,5-diphenyl tetrazolium bromide (MTT) substrate to an insoluble purple formazan product by mitochondrial succinate dehydrogenase enzyme. The MTT enters the cells where it gets reduced to an insoluble, dark purple colored formazan product. Neat and modified rGO-Am dispersions were evaluated in the concentration range from 4 to 1000 µg/mL using an adhesive 96-well plate (n = 3). In the case of films, the round-shaped specimens with a dimeter of 5 mm were glued by BF-6 medical glue (Tula Pharmaceutical Company, Tula, Russia) to the bottom of the nonadhesion 96-well plate (n = 3). Sterilization of glued films was performed by their exposure under a UV light of wide spectrum for 10 min. Next, 100 μL of medium containing 1 × 10^4^ cells was added to each well. The plates were incubated at 37 °C with 5% CO_2_. As a control for dispersions, MG-63 cells were cultured in a culture medium in an adhesive 96-well plate, while pure PCL material was considered as a control for composite films. After 3 days of incubation, MTT (Sigma, St. Louis, MO, USA) solution was added to each well and incubated for 2 h at 37 °C with 5% CO_2_. After the incubation time, the above solution was discarded, and the colored formazan crystals formed were solubilized by adding 50 μL of dimethyl sulfoxide. The absorbance was read at 570 nm in a multiwell plate reader (Thermo Fisher Multiscan Labsystems, Waltham, MA, USA). The absorbance values were plotted using MS Excel software.

## 3. Results and Discussion

### 3.1. Synthesis of rGO-Am

**Figure 1:**
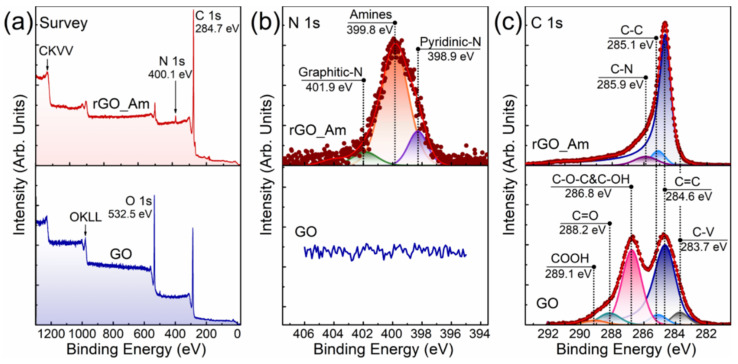
The XPS characterization of initial GO and rGO-Am layers: (**a**) survey spectra, (**b**) high-resolution N 1s spectra and (**c**) high-resolution C 1s spectra.

Figure 1a displays the survey spectra of the GO presented by dominant O 1s and C 1s lines at *hv* = 532.5 eV and *hv* = 284.7 eV with the absence of other spectral features, verifying the purity of the initial material. Upon the amination, the O 1s line substantially diminished, while the N 1s peak at ca. *hv* = 400.1 eV appeared, pointing out the successful introduction of amines by the substitution of the oxygenic moieties in the treated GO. The concentration of nitrogen was estimated to be ca. 3.02 at.%. To further analyze the composition of nitrogen-containing groups embedded upon the applied treatment, high-resolution N 1s spectra were acquired and processed (Figure 1b). Three distinct peaks positioned at *hv* = 398.9 eV, *hv* = 399.8 eV and *hv* = 401.9 eV were discerned, which are related to the presence of pyridines, amines (both primary and secondary), and implemented graphitic nitrogen, respectively [40,41]. As seen, amines are the dominant form of the introduced nitrogen functionalities with their relative content estimated to be up to 88.4%. Accordingly, the concentration of the amines in the synthesized rGO-Am is ca. 2.63 at.%.

The elimination of the oxygenic groups upon the consecutive bromination and amination is pointed out by the C 1s spectra of the GO and rGO-Am (Figure 1c). The initial GO is functionalized by the hydroxyls and epoxides at the basal plane of the graphene layer along with the carbonyls and carboxyls at its edges. The presence of these functional groups is signified by the C–O–C&C–OH, C=O and COOH peaks centered at *hv* = 286.8 eV, *hv* = 288.2 eV and *hv* = 289.1 eV, respectively [42,43]. The C–V peak is related to the nonterminated carbon atoms of the vacancy defects and Stone–Wales defects [44]. The relative concentration of the oxygenic groups and carbon atoms in various states is presented in Table 1, whereas the C/O ratio was calculated to be 2.51. After the amination, all the spectral features related to the oxygenic groups were absent with the retention of only a dominant C=C peak at ~284.6 eV, corresponding to the pristine π-conjugated graphene network, and a C–C peak at ~285.1 eV related to nonconjugated C–C bonds [43]. The concentration of the preserved oxygenic groups is less than 3 at.% with the corresponding C/O ratio of rGO-Am of 32.51 (Table 1). In addition, a C–N peak at ~285.9 eV appears due to the formation of carbon–nitrogen bonds upon the introduction of amines and other nitrogen species [37,45]. Given XPS data on the composition of functional groups and the size distribution of the rGO-Am derived from both the measurements using LD technique and the acquired SEM images of arrays of flakes (Supplementary Materials, Section S1, Figure S1), the molar ratio of the presented amines was estimated. The calculated values are ca. 7.1 mmol/g, which corresponds to 5.4 × 10^9^ amine groups per 1 g of rGO-Am. The details on the calculation of the molar ratio are presented in Supplementary Materials, Section S2.

### 3.2. Modification of rGO-Am with Oligomers of Glutamic Acid

**Figure 2:**
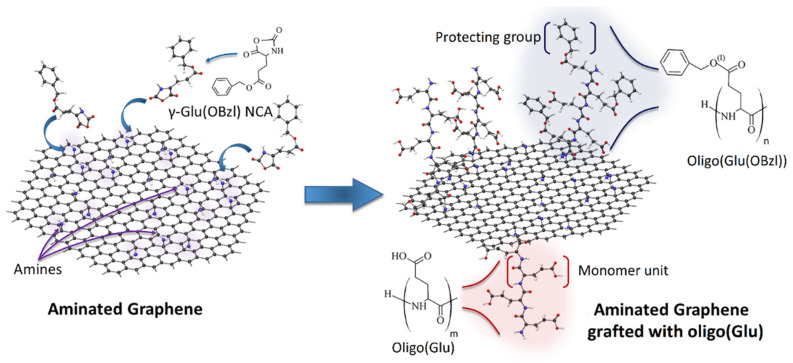
Scheme of rGO-Am modification with oligomers of glutamic acid via ring-opening polymerization of Glu(OBzl) NCA as monomer.

Modification of rGO-Am was carried out using a “grafting from” technique. One of the common methods for the synthesis of oligomers and polymers of amino acids is the ring-opening polymerization of α-amino acid N-carboxyanhydrides (NCAs) catalyzed with the primary amines [46]. In our case, the primary amino groups of rGO-Am played a role of initiating groups for polymerization of γ-protected glutamic acid NCA to prepare the rGO-Am grafted with oligomers of glutamic acid (oligo(Glu)). The scheme of rGO-Am modification with oligo(Glu) is displayed in Figure 2. At first step, the ring-opening polymerization of Glu(OBzl) NCA was carried out to modify rGO-Am. Then, the Bzl protection was removed from γ-carboxylic groups at acidic conditions. The successful grafting of rGO-Am with oligo(Glu) was testified by FTIR spectroscopy and XPS.

**Figure 3:**
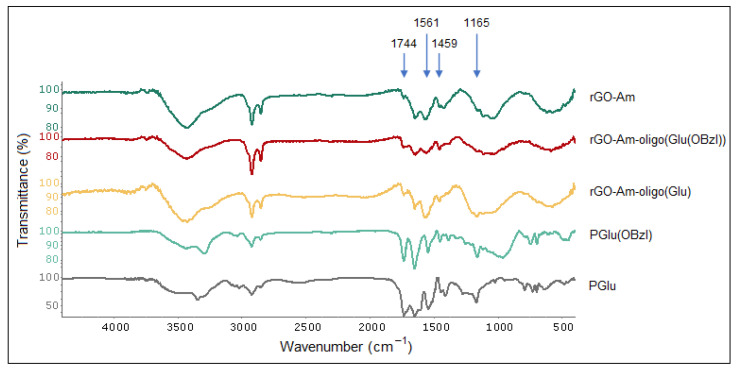
FTIR spectra of neat and modified rGO-Am as well as protected and unprotected poly(glutamic acid) as the standards.

In comparison with neat rGO-Am and protected and unprotected poly(glutamic acid) used as standards, an increase of several characteristic bands corresponding to the groups presented in PGlu was detected in the FTIR spectrum of rGO-Am-oligo(Glu). In particular, an increase in the intensity for the characteristic bands at 1165, 1459, 1561 and 1744 cm^−1^ corresponding to C–N stretching, CH_2_ bending, N–C=O stretching (amid II) and C=O stretching vibrations, respectively, was detected after the grafting of oligo(Glu) from rGO-Am (Figure 3).

**Figure 4:**
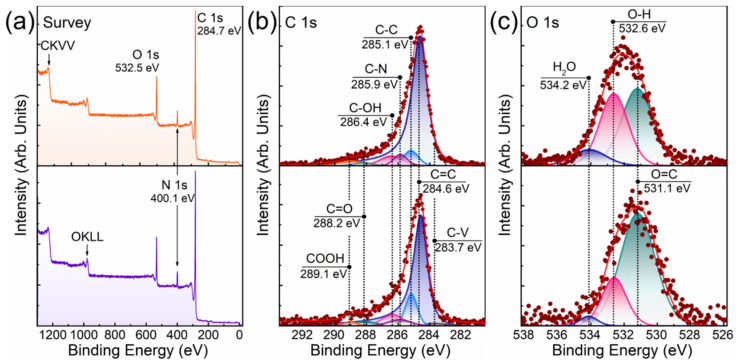
XPS characterization of the rGO-Am grafted with oligo(Glu) (top row) and oligo(Glu(OBzl)) (bottom row): (**a**) survey spectra, (**b**) high-resolution C 1s spectra and (**c**) high-resolution O 1s spectra.

The success of oligo(Glu) grafting from the rGO-Am was also revealed by the corresponding changes in the X-ray photoelectron spectra presented in Figure 4a. In the case of both rGO-Am-oligo(Glu(OBzl)) and rGO-Am-oligo(Glu), the rise of the intensity of the O 1s peak, as well as the N 1s peak, was observed. The quantitative analysis of the acquired survey X-ray photoelectron spectra showed that after the ring-opening polymerization of Glu(OBzl) NCA from rGO-Am, the concentration of the nitrogen increased from 3.02 at.% to ca. 3.52 and 3.47 at.% for protected and unprotected forms, respectively. Given these differences in the nitrogen content and the known chemical structure of a oligo(glutamic acid) (Supplementary Materials, Section S3), the number of oligo(Glu) branches was calculated and estimated to be ca. 2.2 × 10^9^ per 1 g of the obtained material (Supplementary Materials, Section S4). The rate of the functionalization of amines were estimated to be about 4–8 branches and 6–12% of the total number of amines, respectively. It means that the average length of the oligo(Glu) chains is in the range from 4 to 8 monomer units.

The introduction of oligo(Glu) is further signified by the changes of the C 1s spectra displayed in Figure 4b. As seen, after the synthesis of oligo(Glu) the peaks related to carboxyls and carbonyls centered at *hv* = 289.1 eV and *hv* = 288.2 eV, respectively, increased. The appearance of the C–OH peak at *hv* = 286.2 eV related to phenol groups can be also noted, most probably due to the modification of the graphene layer concurrently with the oligo(Glu) growth. Furthermore, the intensity of the C–C peak at *hv* = 285.1 eV increased owing to the presence of nonconjugated C–C bonds in the oligo(Glu) backbone. As one might expect, the most prominent rise of the C–C peak was observed in the case of rGO-Am-oligo(Glu(OBzl)), where the relative content of the carbon in the corresponding state was up to 12.12 at.% (Table 2) [43]. In turn, the relative concentration of the nonconjugated C–C bonds detected for rGO-Am-oligo(Glu) was almost two times lower, i.e., 6.77 at.%. Combined with the redistribution of the relative content of the carboxyls and carbonyls from 1.48 and 3.39 at.% in rGO-Am-oligo(Glu(OBzl)) to 2.84 and 1.65 at.% in rGO-Am-oligo(Glu), respectively, this evolution of the C 1s spectra evidences that, in the latter sample, the protective groups were successfully eliminated from at least 45% of the carboxyl groups in the grafted oligo(Glu). This is also illustrated by the comparative analysis of the O 1s X-ray photoelectron spectra displayed in Figure 4c. In both spectra, three peaks are discerned positioned at *hv* = 531.1 eV, *hv* = 532.5 eV and *hv* = 534.2 eV and attributed to double-bonded oxygen, O–H bonded oxygen, and oxygen in the water molecules, respectively [42,43]. As seen, in the case of rGO-Am-oligo(Glu) the peak of the O–H bonded oxygen related to hydroxyl functionality in the deprotected carboxyl group rises with the increase of its relative concentration from 22.39% to 40.48%. Such changes are commonly observed in the case of moving from carbonylated to carboxylated nanocarbon structures [43,47]. Note that the relative content of the adsorbed water also rises from 3.19% in rGO-Am-oligo(Glu(OBzl)) to 10.83% in rGO-Am-oligo(Glu) upon the elimination of the protecting Bzl groups and, thus, a higher concentration of hydrophilic carboxyl groups. As a net result, the XPS data implies both the successful grafting of oligo(Glu) from rGO-Am and the following deprotection of up to 45% of the Bzl groups from the carboxyls presented in oligo(Glu).

### 3.3. Characterization of Modified rGO-Am

It is known that chemical modifications of materials can change their characteristics and properties [48,49,50]. In order to characterize the modified rGO-Am, such physicochemical methods as DLS and ELS, TGA and DTG were applied for the obtained materials.

According to the DLS and ELS analyses, the aqueous dispersion of neat rGO-Am (pH 7.4) was characterized by the presence of submicron negatively charged particles with a fairly broad size distribution (Table 3). Modification of rGO-Am with protected and unprotected oligomers was accompanied by a decrease in the hydrodynamic diameter of graphene particles due to a reduction of their aggregation. In turn, the modification of rGO-Am with oligo(Glu(OBzl)) was followed by an increase in the surface electrokinetic potential, while, as expected, the deprotection diminished this parameter.

**Figure 5:**
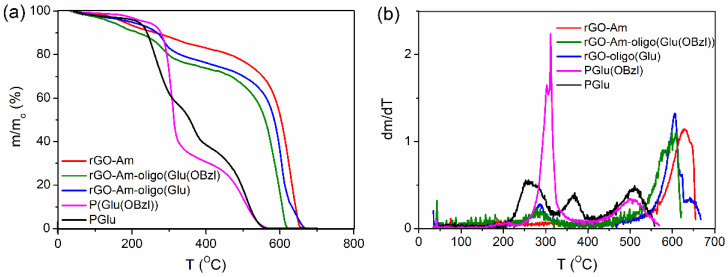
TGA (**a**) and DTG (**b**) curves for the neat and modified rGO-Am as well as protected and unprotected PGlu as standards.

Neat and modified rGO-Am as well as PGlu and PGlu(OBzl) were analyzed by TGA. The obtained TGA and DTG curves are presented in Figure 5. Before the beginning of the region where the destruction of the carbon base of rGO-Am starts (410–420 °C), the mass of the neat rGO-Am consistently falls, starting from 120 to 130 °C (Figure 5a). When the intensive thermal destruction process starts (after 400 °C), the sample has already lost ~18% of the initial mass. This may probably be due to the removal of the residual oxygenic functional groups and solvent retained between the layers of rGO-Am at the early stages of sample heating. In the case of PGlu(OBzl), the main thermal degradation proceeds in two stages: the sample loses ~59% and 29% of its mass in the regions of 240–360 and 420–570 °C, respectively. There is also a low-temperature mass loss in the region of 160–240 °C, where a loss of ~4% of mass is noticeable. These three processes are clearly visible on the differential curve of the sample mass change (Figure 5b). The process of thermal destruction of a PGlu sample proceeds in a significantly different way. In this case, three areas of significant mass drop are clearly visible on the TGA and DTG curves: the sample loses ~38% of its mass in the region of 190–320 °C; the mass falls by ~18% in the region of 330–400 °C; and the sample loses the last ~35% of the mass in the region of 430–560 °C. As can be seen when comparing the differential curves for PGlu(OBzl) and PGlu, only the high-temperature mass loss areas (last peaks on the DTG curve, Figure 5b) coincide for the two compared samples. In turn, rGO-Am-oligo(Glu(OBzl)) and rGO-Am-oligo(Glu) were characterized with a more intensive destruction process in the low-temperature region of 200–360 °C (the maximum intensity of this process is 290 °C) in comparison with neat rGO-Am and PGlu/PGlu(OBzl). Since there are no intense mass losses on the TGA and DTG curves of unmodified rGO-Am in this temperature range, it can be stated that the process observed for the modified samples is the cleavage of oligo(Glu)/oligo(Glu(OBzl)) from rGO-Am and their transition to the gas phase, apparently, with the simultaneous destruction of their oligomer chains. The cleavage of the oligomer fragments from rGO-Am is the most likely due to the identity of the thermodestruction process (the coincidence of temperature regions) for samples containing protected and unprotected oligomer chains. The mass losses for both samples were also very close: ~17% for rGO-Am-oligo(Glu) and 14% for rGO-Am-oligo(Glu(OBzl)). Thus, the mass losses in the region of 200–360 °C may be considered as estimated values of the rGO-Am surface grafting with oligomers of glutamic acid: 14–17%.

**Figure 6:**
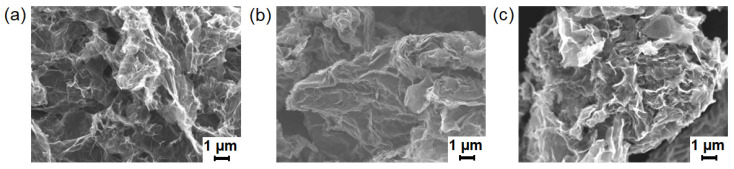
SEM images of rGO-Am platelets on the silicon support (×5000): (**a**) neat rGO-Am, (**b**) rGO-Am-oligo(Glu(OBzl)) and (**c**) rGO-Am-oligo(Glu).

The analysis of the morphology of the neat and modified rGO-Am platelets did not reveal any obvious surface changes (Figure 6). Both materials demonstrate the corrugated structure with a high number of wrinkles and folds of several μm in scale. This results in the reduction of the π–π* interlayer stacking between the layers of rGO-Am, resulting in a highly developed surface of the material and irregular porous network structure. This is in contrast to lamellar GO and rGO platelets, having smoothed surface with a low extent of wrinkling and folding. The corrugation of rGO-Am is an intrinsic characteristic of this material related to both the distortion of the graphene layer at nanoscale due to introduced amines and the interaction of amines with the retained oxygenic groups, resulting in folding at a microscale [34]. Notably, it does not depend on the type of solvent used for the deposition varied from the polar ones (isopropyl alcohol) to nonpolar solvents (trichloromethane and tetrachloromethane). This fact is also supported by the absence of alterations of the rGO-Am morphology after its grafting with both PGlu and PGlu(OBzl), modifying its wetting characteristics.

### 3.4. Manufacturing and Characterization of the Composite Films

In order to prepare the composite materials with rGO-Am and its derivatives, PCL synthesized by ring-opening polymerization of ε-caprolactone was utilized as a polymer matrix in this work. The used PCL had the following characteristics: *M*_w_ = 89,000 and Đ = 1.7 (according to SEC), and η = 0.91 dL/g (according to viscosimetric analysis in CHCl_3_).

The films of pure PCL and its composites with neat and modified rGO-Am were manufactured by casting a polymer solution in CHCl_3_ on the cellophane substrate as described in the experimental part (Section 2.6). After evaporation of chloroform at room temperature, the films were additionally dried to a constant mass at 45 °C to remove solvent traces.

The PCL-based composite films containing neat and modified rGO-Am were tested at room temperature in the uniaxial stretching mode. Pure PCL films were used as a benchmark. During the tests, the following characteristics of the material were determined: the modulus of elasticity (or Young’s modulus) (*E*), tensile strengths (*σ_b_*) and elongation at break (*ε_b_*).

**Figure 7:**
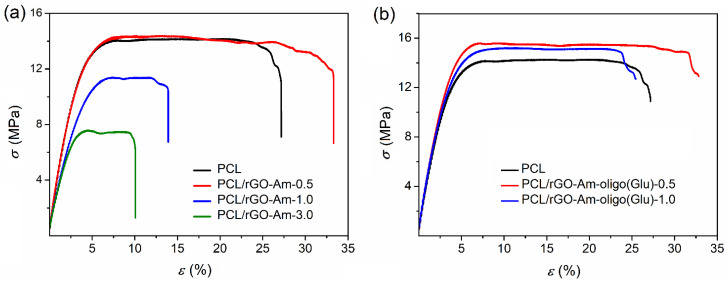
Stress–strain curves for PCL and its composites with neat (**a**) and modifed (**b**) rGO-Am.

It is known that the aggregation of graphene and its derivatives due to the strong π–π interaction prevents the homogeneous distribution of these fillers in the polymer matrix. Recently, it has been shown that grafting of rGO with poly (L-lactic acid) increases the compatibility of this filler with a poly(L-lactic acid)-based matrix [36]. Indeed, the introduction of neat rGO-Am into the PCL matrix led to a sharply negative result. In particular, a noticeable decrease in the Young’s modulus, strength characteristics and deformation resource of the material was observed (Table 4, Figure 7a).

**Figure 8:**
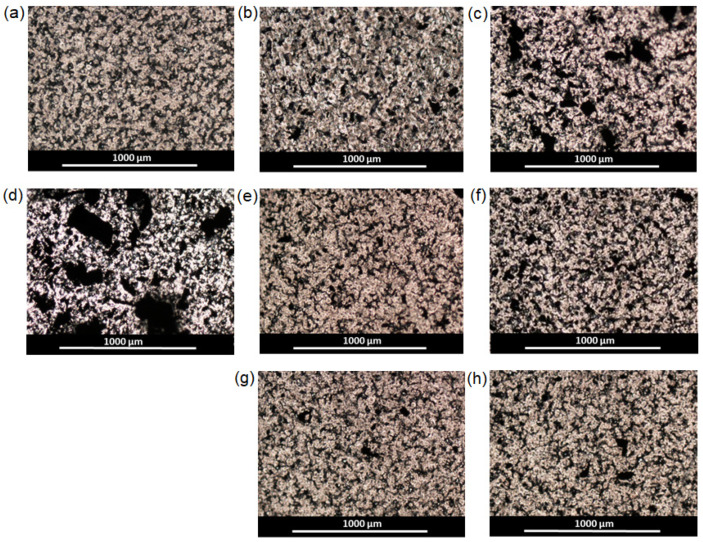
Images of pure PCL and composite films obatined by optical microscopy in transmitted light (×4): (**a**) PCL; (**b**) PCL/rGO-Am-0.5 wt.%; (**c**) PCL/rGO-Am-1.0 wt.%; (**d**) PCL/rGO-Am-3.0 wt.%; (**e**) PCL/rGO-Am-oligo(Glu(OBzl))-0.5 wt.%; (**f**) PCL/rGO-Am-oligo(Glu(OBzl))-1.0 wt.%; (**g**) PCL/rGO-Am-oligo(Glu)-0.5 wt.%; (**h**) PCL/rGO-Am-oligo(Glu)-1.0 wt.%.

**Figure 9:**
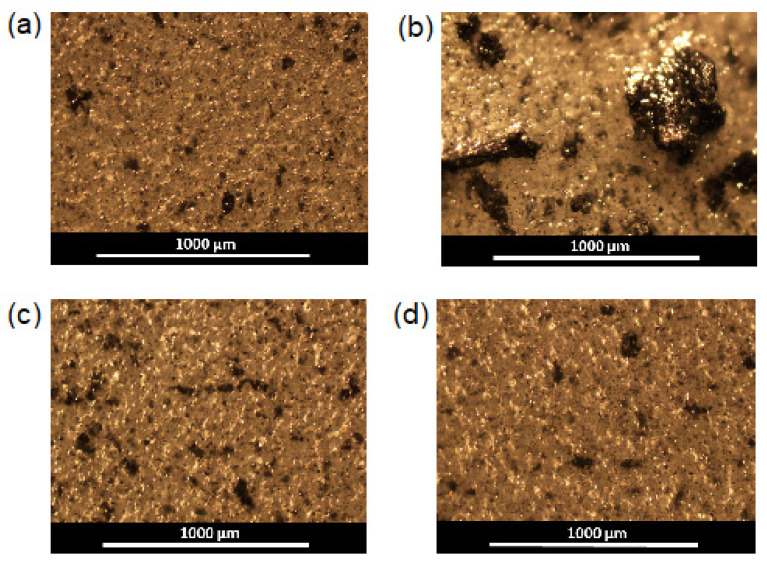
Images of pure PCL and composite films obatined by optical microscopy in reflected light (×4): (**a**) PCL; (**b**) PCL/rGO-Am; (**c**) PCL/rGO-Am-oligo(Glu(OBzl)); (**d**) PCL/rGO-Am-oligo(Glu). The content of the filler in all composites was 1 wt.%.

Moreover, the higher the content of the filler added to the matrix, the worse mechanical properties were detected. The reason for this negative effect is the aggregation of the filler forming large agglomerates in the films. As a consequence, a pronounced heterogeneity of the material structure and the destruction of samples at the phase boundaries took place. The analysis of films by optical microscopy in transmitted light revealed the evident aggregates of rGO-Am in the PCL matrix at filler contents higher than 0.5 wt.% (Figure 8 and Figure 9). At the same time, the modification of rGO-Am with both Bzl-protected and unprotected oligomers of glutamic acid favored a much more uniform distribution of the filler in the PCL matrix due to enhanced compatibility (Figure 8 and Figure 9; Figure S2). In turn, this fact considerably affected the enhancement of the mechanical properties of the manufactured composite materials (Table 4, Figure 7b). The best mechanical properties were established for the PCL-based composite containing 0.5 wt.% of rGO-Am-oligo(Glu) as a filler. A similar trend has been recently observed by Wang et al. for PLA-based films filled with neat GO and GO grafted with PLA [36]. The authors found that composites containing neat GO had poorer mechanical properties than nonfilled PLA. At the same time, the modification of GO with PLA contributed to the enchantment of mechanical properties.

### 3.5. In Vitro Biological Evaluation

**Figure 10:**
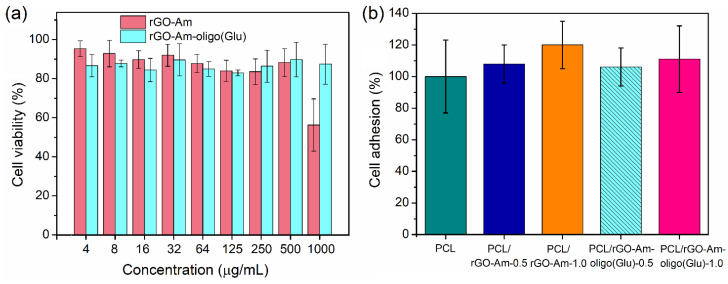
Cytotoxicity study (MTT-test) with MG-63 cells for the dispersions of neat and modified rGO-Am (**a**) as well as PCL-based composite films with rGO-Am and rGO-Am-oligo(Glu) as fillers (**b**).

An in vitro biocompatibility study was carried out with the use of human osteoblast-like cells (MG-63 cell line) for both neat and oligo(Glu)-modified rGO-Am as dispersions and as a part of composite films. Figure 10 illustrates the cell viability in the presence of rGO-Am and rGO-Am-oligo(Glu) taken in the range of concentrations from 4 to 1000 µg/mL. As seen, neat rGO-Am was nontoxic up to the concentration of 500 µg/mL, while rGO-Am-oligo(Glu) was biocompatible in the entire tested concentration range. Thus, the modification of rGO-Am by oligomers of glutamic acid improved the biocompatibility of aminated graphene with living cells.

The high biocompatibility for polymers of glutamic acid with cells was earlier demonstrated in several papers [38,39,51]. Taking into account the high biocompatibility of PCL and low content of the filler in composites, the absence of cytotoxicity for composite films could be expected. Indeed, PCL-based films filled with rGO-Am and rGO-Am-oligo(Glu) demonstrated a similar and comparable level of cell adhesion to the nonfilled PCL films for all tested composite specimens. Therefore, the addition of rGO-Am into PCL, as well as the modification of rGO-Am, did not affect the biocompatibility of PCL with cells.

## 4. Conclusions

In present work, an approach to the modification of aminated graphene by the “grafting from” technique based on ring-opening polymerization of N-carboxyanhydride of glutamic acid γ-benzyl ester was proposed. Grafting of glutamic acid oligomers was testified by both FTIR spectroscopy and XPS. According to XPS analysis, the approximate chain length of grafted oligomers was in the range of 4–8 monomer units. Modification of aminated graphene with oligo(Glu) contributed to a decrease in the average size of rGO-Am particles in the aqueous dispersion. A less pronounced aggregation of modified aminated graphene was also observed after its distribution in the PCL matrix. TGA and DTG analysis of modified aminated graphene revealed its slightly lower thermal stability in comparison with that of neat rGO-Am. However, the mechanical properties of PCL-based composites obtained with neat aminated graphene considerably decreased along with the filler content increase. At the same time, the better distribution of aminated graphene grafted with glutamic acid oligomers improved or preserved the mechanical properties of the composite film. The biocompatibility of PCL/rGO-Am-oligo(Glu) composites with human osteoblast-like cells seems to be very promising for their further use as functional scaffolds for bone tissue regeneration.

## Figures and Tables

**Table 1:** Composition of functional groups (at.%), carbon in different states and C/O ratio estimated from the analysis of the deconvoluted C 1s X-ray photoelectron spectra of GO and rGO-Am.

Component	C–V	C=C	C–C	C–OH and C–O–C/C–OH(p)	C=O	COOH	C–N	C/O Ratio
Binding Energy (eV)	283.7	284.6	285.1	286.8 / 286.4	288.2	289.0	285.9	
GO	5.31	52.94	4.49	28.03	6.68	2.55	-	2.51
rGO-Am	<0.1	89.05	4.93	1.5	<0.6	<0.9	3.02	32.51

**Table 2:** Composition of functional groups (at.%) and C/O ratio estimated from the analysis of the deconvoluted C 1s X-ray photoelectron spectra.

Component	C–V	C=C	C–C	C–OH and C–O–C/C–OH(p)	C=O	COOH	C–N	C/O Ratio
Binding Energy (eV)	283.7	284.6	285.1	286.8 / 286.4	288.2	289.0	285.9	
rGO-Am-oligo(Glu(OBzl))	1.93	69.55	12.12	8.01	3.39	1.48	3.52	6.87
rGO-Am-oligo(Glu)	<0.1	78.79	6.77	6.48	1.65	2.84	3.47	7.38

**Table 3:** Characteristics of the neat and modified rGO-Am particles in aqueous solution (pH 7.4) determined by dynamic and electrophoretic light scattering.

Sample	D_H_ (nm)	PDI	ζ-potential (mV)
rGO-Am	465 ± 71	0.56	−38.4 ± 7.1
rGO-Am-oligo(Glu(OBzl))	369 ± 66	0.42	−32.1 ± 5.6
rGO-Am-oligo(Glu)	302 ± 49	0.52	−40.0 ± 5.1

**Table 4:** Mechanical properties of PCL and its composites with neat and modified rGO-Am.

Specimen	Filler Content (%)	E (MPa)	σ _b_ (MPa)	ε _b_ (%)
PCL	-	396 ± 13	13.9 ± 0.5	25 ± 2
PCL/rGO-Am	0.5	369 ± 43	13.6 ± 0.3	25 ± 3
	1.0	348 ± 32	11.4 ± 0.5	12 ± 1
	3.0	270 ± 28	7.5 ± 0.6	10 ± 1
PCL/rGO-Am-oligo(Glu(OBzl)	0.5	415 ± 20	13.5 ± 0.5	21 ± 2
	1.0	396 ± 32	13.1 ± 0.4	15 ± 2
PCL/rGO-Am-oligo(Glu)	0.5	434 ± 39	14.8 ± 0.4	27 ± 2
	1.0	444 ± 17	14.6 ± 0.4	23 ± 2

## Data Availability

The data are contained within the article and supplementary materials.

## Supplementary Materials

The following are available online at https://www.mdpi.com/article/10.3390/polym13162628/s1, Figure S1: The analysis of the size distribution of rGO-Am. (a) The size distribution histogram derived from the laser diffraction measurements. SEM images of (b) an array and (c) a single flake of rGO-Am; S1. Calculations of the molar ratio and number of functional groups in rGO-Am; S2. Calculations of the oligo(Glu) content after the grafting; Figure S2. SEM images of nonfilled PCL and its composite films (×100).
