# Supplementary material for: Aminated Graphene-Graft-Oligo(Glutamic Acid) /Poly(ε-Caprolactone) Composites: Preparation, Characterization and Biological Evaluation
Source: Polymers (Basel). 2021 Aug 7;13(16):2628. doi: 10.3390/polym13162628 (PMC8401938; doi:10.3390/polym13162628)
Supplement: Supplementary file 1 [file polymers-13-02628-s001.zip › polymers-1322033-supplementary.pdf]

Supplementary Materials

Aminated Graphene-graft-Oligo(Glutamic Acid)/ Poly(ε-Caprolactone) Composites: Preparation, Characterization and Biological Evaluation

Mariia Stepanova[1], Olga Solomakha[1], Maxim Rabchinskii[2]*, Ilia Averianov[1], Iosif Gofman[1], Yuliya Nashchekina[3], Grigorii Antonov[2], Aleksey Smirnov[2], Boris Ber[2], Aleksey Nashchekin[2] and Evgenia Korzhikova-Vlakh[1]*

1 Institute of Macromolecular Compounds, Russian Academy of Sciences, St. Petersburg 199004, Russia
2 Ioffe Institute, Politekhnicheskaya st. 26, St. Petersburg, 194021, Russia
3 Institute of Cytology, Russian Academy of Sciences, St. Petersburg 194064, Russia

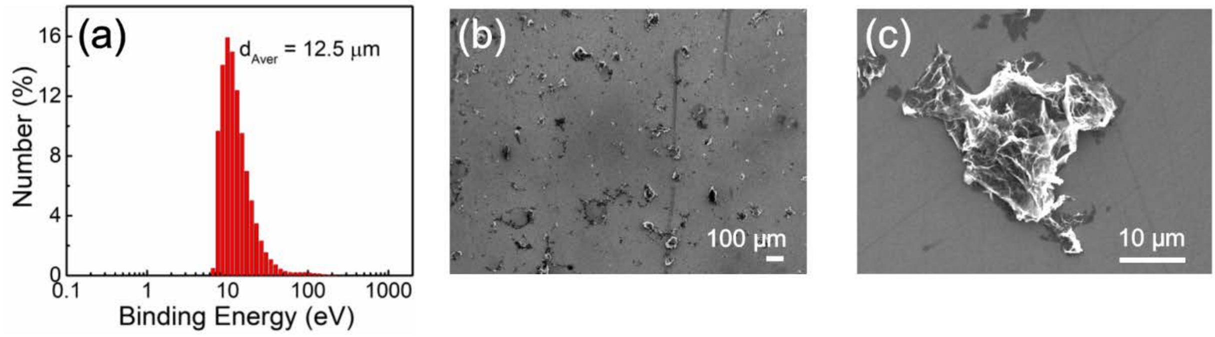

Figure S1. The analysis of the size distribution of rGO-Am. (a) The size distribution histogram derived from the Laser Diffraction measurements. SEM images of (b) array and (c) a single flake of rGO-Am.

S1. Calculations of the molar ratio and number of functional groups in rGO-Am

To calculate the molar ratio of the amines as well as other oxygenic and nitrogen functionalities the following relation was used:

$$\nu = \frac{m_{rGO-Am} * N_A}{m_{Flake} * N_{group}} \tag{1}$$

where m_{rGO-Am} is the total mass of the rGO-Am, N_A is the Avogadro constant, m_{Flake} is the mass of a single flake and N_{group} is the total number of the functional groups, for which the molar ratio is calculated – amines in our case. To estimate the mol/g ratio the m_{rGO-Am} is taken as 1 g, whereas the m_{Flake} and N_{group} are the calculated values. The former value is estimated from the following relation:

$$M_{flake} = M_{hex} * N_{hex} \tag{2}$$

where M_{hex} is the mass of one hexagon and N_{hex} is the number of hexagons that make up the flake. The mass of one hex can be calculated as follows:

$$M_{hex} = 6 * Da(C) * \frac{1}{3} + (\sum Da_{fg} * at\%) \tag{3}$$

where Da_{fg} is Dalton units of atoms of functional groups, also known as unified atomic mass unit, at% - is the relative content of the functional groups determined from the processed C 1s and N 1s X-ray photoelectron spectra.

The N_{hex} is calculated as follows:

$$N_{hex} = S_{fl}/S_{hex} \tag{4}$$

The S_{fl} refers to the mean area of the flakes estimated from the size distribution determined experimentally using laser diffraction, whereas S_{hex} is calculated as:

$$S_{hex} = 1{,}5 * \sqrt{3} * a \quad (5)$$

where a is the length of a single C=C bond in the graphene network, equal to 0.142 nm.
In turn, the N_{group} can be estimated by the following relation:

$$N_{group} = N_{hex} * at\% \quad (6)$$

Given these relations, the atomic concentration of the functional groups from the XPS data (Table 1), the average size of rGO-Am flakes of 12.5 μm (Section S1) and the $Da_{NH3} = 17$ the molar ratio of amines was calculated to be 7.1 mmol/g, whereas their number in 1 g of rGO-Am is estimated to be ca. 5.4×10^{9}.

S2. Calculations of the oligo(Glu) content after the grafting

To estimate the content of oligomer chains in the rGO-Am after the polymerization the XPS data was processed as follows. To quantify the relative content of the certain element, the following relation is commonly used:

$$C_x = \frac{\frac{S_x}{I_x}}{\sum_a \frac{S_a}{I_a}} * 100 \quad (7)$$

where **C_x** – is the atomic concentration of a certain element (at%), **S_x** is the area of the peak of the element analyzed, **I_x** – is the relative sensitivity factor (RSF), Σ is the sum of the areas of all the peaks divided to the corresponding RSF values. The areas of the peaks are presented in arbitrary units. Using this formula, the elemental composition of the rGO-Am layer was calculated from the corresponding survey spectrum, giving the relative content of C, O, and N of ca. 92 at%, 5 at%, and 3 at%.

Note that the **S_x** is proportional to the number of atoms of the corresponding element and basically the Eq. 7 can be re-written in the form of:

$$C_x = \frac{N_x}{N_{tot}} * 100 \quad (8)$$

where **N_{tot}** is the total number of atoms in a measured area of the sample during the XPS studies dependent on the area of the probing beam, the penetration depth of the X-ray radiation with the chosen energy, its chemistry. This value is hardly estimated but in the case of using the same experimental setup at the same conditions the **N_{tot}** value changes only due to the variation of the chemical composition of the sample – since the parameters of the probing beam keep constant. Thus, upon the rGO-Am grafting by oligo(Glu) the **N_{tot}** value will change only due to the changes in the chemistry of the sample that is the addition of the oligo(Glu) chains to the aminated graphene. In this case, N_{tot} can be written in the form of:

$$N_{tot}\left(Am_{Oligo(Glu)}\right) = N_{tot}(Am) + N(Oligo(Glu)) \quad (9)$$

whereas the N_x is:

$$N_x\left(Am_{Oligo(Glu)}\right) = N_x(Am) + N_x(Oligo(Glu)) \quad (10)$$

One branch of oligo(Glu) molecule with a protecting group is presented by 12 C atoms, 1 nitrogen atom, and 3 oxygen atoms. The total number of all the atoms constituting a single oligo(Glu) is dependent on the number of branches **M**. At the same time, the number of oligo(Glu) molecules is dependent on the and the efficiency of the polymerization denoted hereinafter as $\boldsymbol{\gamma}$ and related to the relation between the number of amine groups grafted by oligo(Glu) and the total number of amines. Thus, N(oligo(Glu)) can be written in the form:

$$N_{tot}(Oligo(Glu)) = [12 + 1 + 3] * M * N_{NH_3} * \gamma \quad (11)$$

where N_{NH_3} is the number of amine groups derived from the Rel. 8 as

$$N_{NH_3} = C_{Nitrogen}(Am) * N_{tot}(Am) * 100 \quad (12)$$

In a same way the number of carbons, nitrogen or oxygen atoms (N_x) can be expressed in a form of:

$$N_x(Oligo(Glu)) = A * M * N_{NH_3} * \gamma \quad (13)$$

where A is equal to 12, 1, and 3 for the carbon, nitrogen, or oxygen, that is the number of the corresponding element in the one branch of oligo(Glu).

Combining the relations 8-13 the final relation, determining the C_x value for a certain element in the Am-oligo(Glu) can be derived:

$$C_x = \frac{[C_x * N_{tot}(Am) * 100] + [A * M * \gamma * (C_{Nitrogen}(Am) * N_{tot}(Am) * 100)]}{N_{tot}(Am) + \{[12+1+3] * M * \gamma * (C_{Nitrogen}(Am) * N_{tot}(Am) * 100)\}} * 100 \quad (14)$$

As seen, the $\mathbf{N_{tot}}$ is eliminated, whereas the C_x value corresponds to the at.% of a certain element determined by means of XPS. For instance, in the case of nitrogen, the Rel.14 will be modified into a form:

$$C_{N(at\%)}(Am_{Oligo(Glu)}) = \frac{[C_{N(at\%)}(Am) * 100] + [A * M * \gamma * C_{N(at\%)}(Am) * 100)]}{N_{tot}(Am) + \{[12+1+3] * M * \gamma * C_{N(at\%)}(Am) * 100)\}} * 100 \quad (15)$$

Given this relation and the experimentally determined concentrations of nitrogen in rGO-Am and Am- oligo(Glu) samples, 3.02 at%, and 3.52 at%, respectively, the maximum total number of oligo(Glu) branches that is the product of M and γ in the rGO-Am-oligo(Glu) sample can be estimated. The calculated value is 0.4 ratio of the number of amines in the rGO-Am or, considering the number of amines in rGO-Am (Section S2), ca. 2.172×10^9 per 1 g. Since both M and γ cannot be reliably determined, their exact values were not calculated. However, Rel. 15 allows one to determine one of these values assigning a second one.

PCL | **PCL + 0.5 wt% rGO-Am** | **PCL + 0.5 wt% rGO-Am-oligo(Glu)**

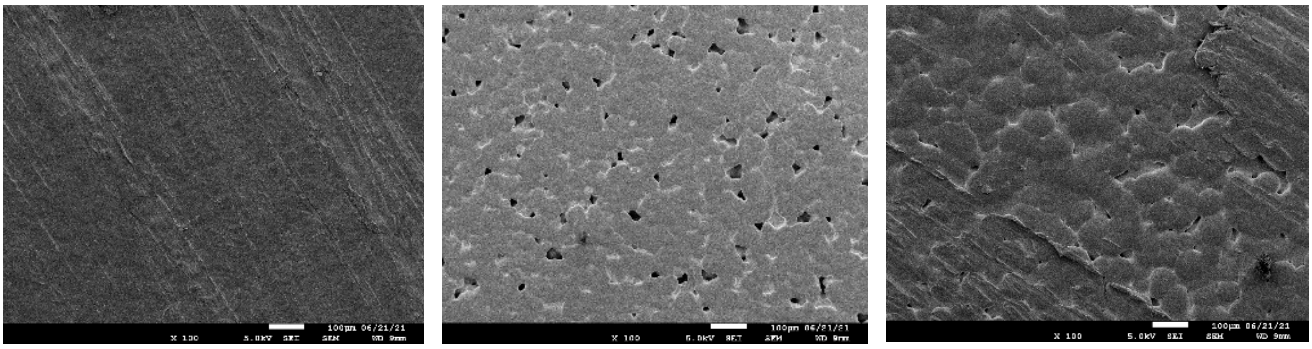

Figure S2. SEM images of non-filled PCL and its composite films (×100).
